# Trastuzumab deruxtecan for the treatment of patients with HER2-positive breast cancer with brain and/or leptomeningeal metastases: an updated overall survival analysis using data from a multicenter retrospective study (ROSET-BM)

**DOI:** 10.1007/s12282-024-01614-1

**Published:** 2024-08-12

**Authors:** Takahiro Nakayama, Naoki Niikura, Takashi Yamanaka, Mitsugu Yamamoto, Kazuo Matsuura, Kenichi Inoue, Sachiko Takahara, Hironori Nomura, Shosuke Kita, Miki Yamaguchi, Tomoyuki Aruga, Nobuhiro Shibata, Akihiko Shimomura, Yuri Ozaki, Shuji Sakai, Daisuke Takiguchi, Takehiko Takata, Armin Bastanfard, Kazuhito Shiosakai, Junji Tsurutani

**Affiliations:** 1 Department of Breast and Endocrine Surgery, Osaka International Cancer Institute, Osaka, Japan; 2 Department of Breast Oncology, Tokai University School of Medicine, 143 Shimokasuya, Isehara, Kanagawa 259-1193 Japan; 3 Department of Breast Surgery and Oncology, Kanagawa Cancer Center, Kanagawa, Japan; 4 Department of Breast Oncology, Hokkaido Cancer Center, Hokkaido, Japan; 5 Department of Breast Oncology, Saitama Medical University International Medical Center, Saitama, Japan; 6 Division of Breast Oncology, Saitama Cancer Center, Saitama, Japan; 7 Department of Breast Surgery, Kitano Hospital, Osaka, Japan; 8 First Department of Surgery, University of the Ryukyus Hospital, Okinawa, Japan; 9 Department of Medical Oncology, National Cancer Center Hospital, Tokyo, Japan; 10 Department of Breast Surgery, JCHO Kurume General Hospital, Fukuoka, Japan; 11 Department of Breast Surgery, Tokyo Metropolitan Cancer and Infectious Diseases Center Komagome Hospital, Tokyo, Japan; 12 Cancer Treatment Center, Kansai Medical University Hospital, Osaka, Japan; 13 Department of Breast and Medical Oncology, National Center for Global Health and Medicine, Tokyo, Japan; 14 Department of Breast Oncology, Aichi Cancer Center, Aichi, Japan; 15 Department of Diagnostic Imaging and Nuclear Medicine, Tokyo Women’s Medical University, Tokyo, Japan; 16 Oncology Medical Science Department I, Daiichi Sankyo Co., Ltd., Tokyo, Japan; 17 Data Intelligence Department, Daiichi Sankyo Co., Ltd., Tokyo, Japan; 18 Advanced Cancer Translational Research Institute, Showa University, Tokyo, Japan

**Keywords:** Trastuzumab deruxtecan, HER2+, Breast cancer, Brain metastasis, Leptomeningeal

## Abstract

We provide updated results (median follow-up duration: 20.4 months) of a retrospective study on the effectiveness of trastuzumab deruxtecan (T-DXd) in patients with human epidermal growth factor receptor 2-positive (HER2+) breast cancer with brain metastases (BM) and/or leptomeningeal disease (ROSET-BM). Median progression-free survival (PFS) was 14.6 months. Median overall survival (OS) was not reached (NR); 24-month OS rate was 56.0%. Subgroup analysis showed that median PFS was 13.2 months in patients with analytical active BM, 17.5 months in patients with leptomeningeal carcinomatosis (LMC), and NR in patients with analytical stable BM (24-month PFS rates in patients with analytical active BM, LMC, and analytical stable BM were 32.7%, 25.1%, and 60.8%, respectively). Median OS was 27.0 months in patients with analytical active BM and NR in patients with LMC or analytical stable BM (24-month OS rates in patients with analytical active BM, LMC, and analytical stable BM were 52.0%, 61.6%, and 71.6%, respectively). The most common adverse event leading to discontinuation of T-DXd was interstitial lung disease (ILD; 23.1%); median ILD onset time among patients who discontinued T-DXd treatment due to ILD was 5.3 months. T-DXd has promising effectiveness in heavily pre-treated HER2+ metastatic breast cancer patients with BM and LMC. The incidence and median onset time of ILD were similar to those of Japanese subgroups in previous studies.

**Supplementary Information:**

The online version contains supplementary material available at 10.1007/s12282-024-01614-1.

## Introduction

A recent review and meta-analysis reported that the incidence of brain metastases (BM) in metastatic breast cancer (MBC) patients is higher in patients with human epidermal growth factor receptor 2-positive (HER2+) and triple negative breast cancer than in those with hormone receptor positive/HER2 negative breast cancer (31% and 32% vs 15%, respectively) [1]. Breast cancer subtype also affects the prognosis of BM [1]. Recently, systemic drug therapy that includes anti-HER2 drugs has become an option for HER2+ breast cancer patients with BM who are eligible for systemic therapy [1].

Previously, the results from a multicenter, retrospective, medical chart review study (ROSET-BM) suggested robust effectiveness of trastuzumab deruxtecan (T-DXd) in patients with HER2+ breast cancer with BM in real world practice [2]. The median follow-up duration was 11.2 months (95% confidence interval [CI] 10.2, 12.5). Median overall survival (OS) was not reached (NR) (95% CI 16.1, -), 12-month OS rate was 74.9%, median progression-free survival (PFS) was 16.1 months (95% CI 12.0, -), and median time-to-treatment failure (TTF) was 9.7 months (95% CI 6.3, 13.0). The median number of prior lines of therapy was 4 (range 1–15). These results from this primary study were cited in the Japanese Breast Cancer Society Clinical Practice Guidelines for systemic treatment of breast cancer (2022 edition) [3].

This report provides 1 year updated data, including OS, PFS, and TTF data with a longer follow-up (median follow-up duration, 20.4 months [95% CI 16.4, 22.5]).

## Patients and methods

### Study design and patients

The study design and patient inclusion/exclusion criteria have been described previously [2]. Briefly, this was a multicenter, retrospective, medical chart review study (UMIN-CTR identifier number: UMIN000044995). For this updated OS analysis, the data cutoff for survival and other information was October 31, 2022. Data entry began on November 1, 2022, and information from medical records was entered retrospectively.

This study was conducted in accordance with the Declaration of Helsinki and adhered to local ethical guidelines. Informed consent from patients was waived per local ethical guidelines.

### Outcomes

OS, PFS, TTF, and time-to-discontinuation of T-DXd treatment due to interstitial lung disease (ILD) were evaluated in the total population. The relationship between background factors of poor prognosis of BM and OS was also evaluated.

### Analytical active BM subgroup

Using Independent Central Review (ICR), patients with tumor growth according to two brain imaging comparisons before T-DXd administration were defined as “active by ICR.” In this study, patients who had not undergone whole-brain radiotherapy within 30 days before T-DXd administration and who did not have leptomeningeal carcinomatosis (LMC) were categorized as “analytical active BM.”

### Analytical stable BM subgroup

Patients who were not classified as active and/or having LMC by ICR were defined as “stable by ICR.” In this study, patients who were defined as stable by ICR and who had irradiated active BM were categorized as “analytical stable BM.”

### LMC subgroup

Patients judged to have LMC by ICR were classified as “active with LMC” or “only LMC” and were categorized as “LMC” in the analysis.

### Statistical methods

The sample size was based on the number of cases that could be collected within the study time frame after a preliminary survey at the institutions that would be participating. Median OS, PFS, TTF, and time-to-discontinuation of T-DXd treatment among patients with ILD and their corresponding 95% CIs were calculated using the Kaplan–Meier method. Survival probabilities (12, 18, and 24 months) and their 95% CIs were calculated. Subgroup analyses were performed by BM classification (active, stable, and LMC). Multivariate stepwise analysis was performed on OS. Variables were based on components of Graded Prognostic Assessment (GPA) scoring (not Karnofsky performance status [PS]) and included 10 factors of clinical importance from a medical perspective, such as age (≥ 60 vs < 60 years), number of BM (1 vs ≥ 2), metastasis except brain (negative vs positive), and Eastern Cooperative Oncology Group (ECOG) PS (0–1 vs ≥ 2). A stepwise Cox proportional hazards regression model was used for the multivariate analysis, and variables entered into the model if *P* = 0.20 [4]. Unless otherwise stated, the 5% significance level and 95% CI were two-sided.

## Results

**Fig. 1:**
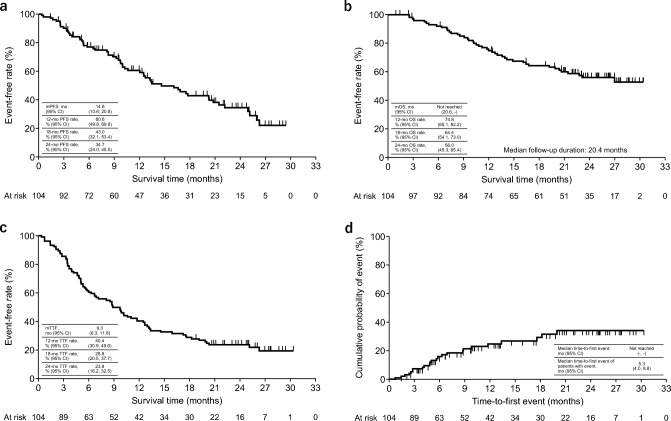
**a** PFS, **b** OS, **c** TTF, and **d** time-to-discontinuation of T-DXd treatment due to ILD (total population). *CI* confidence interval, *ILD* interstitial lung disease, *mo* month, *mOS* median overall survival, *mPFS* median progression-free survival, *mTTF* median time-to-treatment failure, *OS* overall survival, *PFS* progression-free survival, *T-DXd* trastuzumab deruxtecan, *TTF* time-to-treatment failure

Baseline patient characteristics are summarized in Table 1. The median time from first diagnosis of MBC to first administration of T-DXd was 37.5 (range 1.7–256) months, and median time from first diagnosis of BM to first administration of T-DXd was 18.9 (0.1–129) months. Median PFS was 14.6 (95% CI 10.6, 20.8) months, median OS was NR (95% CI 20.6, -), 24-month OS rate was 56.0% (95% CI 45.3, 65.4), and median TTF was 9.3 (95% CI 6.3, 11.8) months (Fig. 1a–c). In total, 24 of the 104 patients (23.1%) discontinued their T-DXd treatment because of ILD, and median time-to-discontinuation of T-DXd due to ILD was 5.3 (95% CI 4.0, 8.8) months (Fig. 1d). The incidence of Grade 1 ILD was higher than ILD of other grades (n [%]: Grade 1, 14 [13.5%]; Grade 2, 3 [2.9%]; Grade 3, 5 [4.8%]; Grade 4, 2 [1.9%]; Grade 5, 0 [0.0%]). Table 2 summarizes the primary results and results from an additional 1 year of follow-up.

**Table 1:** Baseline characteristics (total population)

Characteristic	*N* = 104
Sex	
Male/female	1 (1.0)/103 (99.0)
Age, years	
< 65/ ≥ 65	75 (72.1)/29 (27.9)
HER2 status (IHC)^a^	
0, 1+/2+/3+	0 (0.0)/18 (17.3)/84 (80.8)
Unknown	2 (1.9)
HER2 status (ISH)	
Positive/negative	29 (27.9)/1 (1.0)
Unknown	74 (71.2)
Estrogen receptor status	
Positive/negative	59 (56.7)/44 (42.3)
Unknown	1 (1.0)
Progesterone receptor status	
Positive/negative	43 (41.3)/61 (58.7)
Unknown	0 (0.0)
Surgery for primary breast cancer	71 (68.3)
Number of prior therapies for MBC	
0–2	25 (24.0)
≥ 3	79 (76.0)
Median (Q1, Q3)	4.0 (3.0, 7.0)
Prior treatment for MBC	
Trastuzumab	94 (90.4)
Pertuzumab	88 (84.6)
Trastuzumab emtansine	91 (87.5)
Lapatinib	37 (35.6)
Time from first diagnosis of BM to first administration of T-DXd, months, median (range)	18.9 (0.1–129)
Time from first diagnosis of MBC to first administration of T-DXd, months, median (range)	37.5 (1.7–256)
ECOG PS	
0/1/2/3–4	27 (26.0)/54 (51.9)/12 (11.5)/4 (3.8)
Unknown	7 (6.7)
Visceral metastasis except the brain	79 (76.0)
Clinical presentation of BM	
Symptomatic	32 (30.8)
Asymptomatic	72 (69.2)
Drug used for symptoms of BM	
Steroids	15 (14.4)
Anti-epileptics	11 (10.6)
Local treatment for BM^b^	
Treated	99 (95.2)
Whole-brain radiation	56 (53.8)
Within 30 days	6 (5.8)
Stereotactic irradiation	64 (61.5)
Surgery to remove a tumor	27 (26.0)
Untreated	5 (4.8)
Classification of BM by ICR	
Active BM	90 (86.5)
Without LMC	73 (70.2)
With LMC	17 (16.3)
Stable BM	6 (5.8)
Only LMC	2 (1.9)
Image not classified	6 (5.8)
Definition of analytical BM and LMC subgroups	
Analytical active BM	67 (64.4)
Analytical stable BM	12 (11.5)
LMC	19 (18.3)
Image not classified	6 (5.8)
Number of BM	
1	18 (17.3)
2–4	28 (26.9)
5–9	17 (16.3)
≥ 10	27 (26.0)
Brain images not submitted after T-DXd administration	14 (13.5)
Size of BM, cm (*n* = 55)	
Mean ± SD	2.1 ± 0.9
Karnofsky PS	
0–40	3 (2.9)
50–70	22 (21.2)
80–100	45 (43.3)
Unknown	34 (32.7)
GPA score	
0–1	0 (0.0)
1.5–2.0	9 (8.7)
2.5–3.0	43 (41.3)
3.5–4.0	18 (17.3)
Unknown	34 (32.7)

Data are no. (%), unless otherwise stated

*BM* brain metastasis, *CNS* central nervous system, *ECOG* Eastern Cooperative Oncology Group, *GPA* Graded Prognostic Assessment, *HER2* human epidermal growth factor receptor 2, *ICR* Independent Central Review, *IHC* immunohistochemistry, *ISH* in situ hybridization, *LMC* leptomeningeal carcinomatosis, *MBC* metastatic breast cancer, *PS* performance status, *Q* quartile, *SD* standard deviation, *T-DXd* trastuzumab deruxtecan

^a^HER2 status was based on the primary tumor. Two patients with IHC unknown were ISH + . One patient was IHC 2+ and ISH − , but the brain lesion removed by surgery was IHC 3+

^b^Includes patients who have received multiple local treatments

Created from Niikura N, Yamanaka T, Nomura H, Shiraishi K, Kusama H, Yamamoto M, et al. npj Breast Cancer 2023;9:82; https://doi.org/10.1038/s41523-023-00584-5

**Table 2:** Summary of results

Variable	Primary results; October 2021 data cut off (*N* = 104)	Updated results; October 2022 data cutoff (*N* = 104)
Median follow-up duration, months (95% CI)	11.2 (10.2, 12.5)	20.4 (16.4, 22.5)
Median OS, months(95% CI)	NR (16.1, -)	NR (20.6 , -)
12-month OS rate, %	74.9	74.8
24-month OS rate, %	N/A	56.0
Median PFS, months (95% CI)	16.1 (12.0, -)	14.6 (10.6, 20.8)
Median TTF, months (95% CI)	9.7 (6.3, 13.0)	9.3 (6.3, 11.8)
Discontinued due to ILD, %	18.3	23.1

*CI* confidence interval, *ILD* interstitial lung disease, *N/A* not applicable, *NR* not reached, *OS* overall survival, *PFS* progression-free survival, *TTF* time-to-treatment failure

**Fig. 2:**
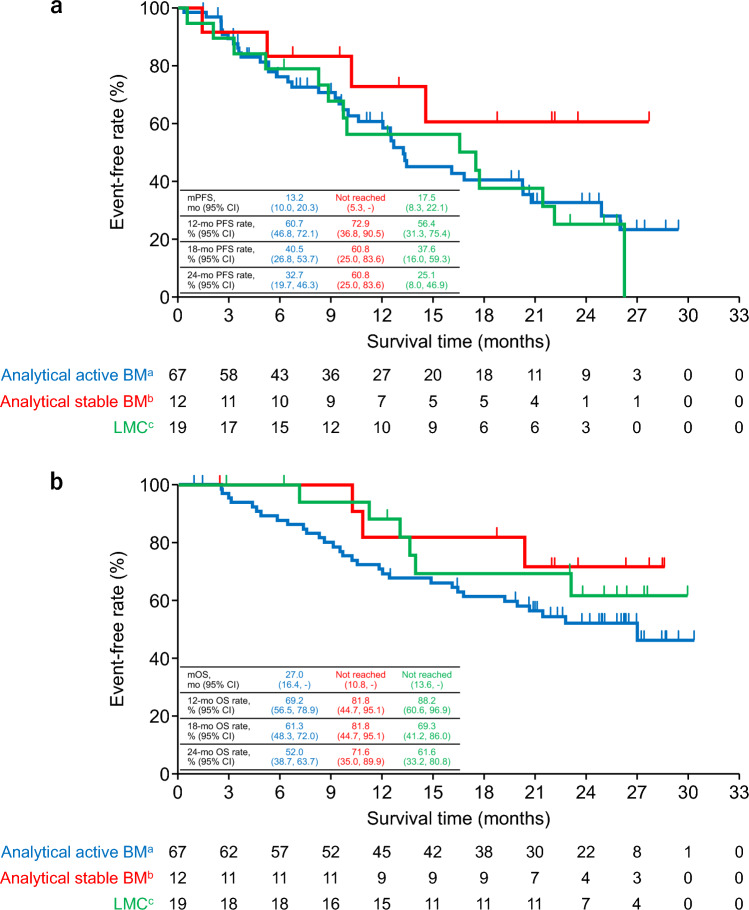
**a** PFS and **b** OS by classification of BM. ^a^Active (not including WBRT within 30 days). ^b^Stable + active with WBRT within 30 days. ^c^Active with LMC/LMC only. *BM* brain metastasis, *CI* confidence interval, *LMC* leptomeningeal carcinomatosis, *mo* month, *mOS* median overall survival, *mPFS* median progression-free survival, *OS* overall survival, *PFS* progression-free survival, *WBRT* whole-brain radiotherapy

BM was classified as analytical active BM, analytical stable BM, and LMC by ICR, and PFS and OS calculated for each subgroup (Fig. 2). Median PFS was 13.2 (95% CI 10.0, 20.3) months in patients with analytical active BM, NR (95% CI 5.3, -) in patients with analytical stable BM, and 17.5 (95% CI 8.3, 22.1) months in patients with LMC. The 24-month PFS rates in patients with analytical active BM, analytical stable BM, and LMC were 32.7%, 60.8%, and 25.1%, respectively. Median OS was 27.0 (95% CI 16.4, -) months in patients with analytical active BM and NR in patients with analytical stable BM (95% CI 10.8, -) or LMC (95% CI 13.6, -). The 24-month OS rates in patients with analytical active BM, analytical stable BM, and LMC were 52.0%, 71.6%, and 61.6%, respectively.

Neither the univariate nor the multivariate stepwise analysis for OS identified any significant variables (Online Resource Supplementary Table 1).

## Discussion

This study investigated 104 HER2+ breast cancer patients with BM treated with T-DXd in a real-world clinical setting. The findings indicate the potential of T-DXd for treating this population, including patients with analytical active, analytical stable BM, and LMC.

We found that 24-month OS rate was 56.0% (median OS was NR), and median PFS was 14.6 months among all patients with BM treated with T-DXd. In the subgroup analyses, the median OS was 27.0 months in patients with analytical active BM, and the 24-month OS rates in patients with LMC and analytical stable BM were 61.6% and 71.6%, respectively (median OS were NR in patients with LMC or stable BM). Prior to T-DXd approval in Japan, the studies KBCSG-TR 1917 [5] and WJOG12519B [6] reported on the effectiveness of post-trastuzumab emtansine treatment in HER2+ MBC patients with BM in a real-world setting. Among patients with BM, the median PFS and OS were 5.7 and 16.1 months, respectively, in the KBCSG-TR 1917 study [5], and 4.9 and 16.4 months, respectively, in the WJOG12519B study [6]. In the present study with 1 year follow-up, the effectiveness of T-DXd was demonstrated by stable PFS beyond 12 months and OS beyond 24 months.

Recently, evidence has emerged that T-DXd is effective for BM (TUXEDO-1, DEBBRAH, DESTINY-Breast01, 02, 03 trials) [7–9]. In the TUXEDO-1 trial, 14 patients with active HER2+ breast cancer with BM had received a median of 2 prior treatment lines and 60% had progressive BM. At a median follow-up of 26.5 months, median PFS was 21 months and median OS was NR [7]. In the DEBBRAH study, at the median treatment duration of 9.0 months, median PFS was 8.9 months and median OS was 13.3 months [8]. According to a pooled analysis of T-DXd in patients with HER2+ MBC with BM from DESTINY-Breast01, 02, and 03, of 148 patients with BM at baseline who received T-DXd, 104 (70.3%) had treated BM and 44 (29.7%) had untreated BM. Patients had a median of 3 prior regimens in the metastatic setting. Median treatment duration was 12.7 months with T-DXd and 5.6 months with a comparator. Numerically longer median central nervous system PFS was observed in patients with treated/stable and active BM randomized to T-DXd vs a comparator (stable BM: 12.3 vs 8.7 months, active BM: 18.5 vs 4.0 months) [9]. Given these results, our findings suggest that T-DXd is a treatment option for HER2+ breast cancer patients with BM, including those with LMC and active and stable BM.

In the additional 1 year follow-up period to the previous analysis, the incidence of ILD increased by 5 cases, but most were low-grade events (Grade 1, 4 cases; Grade 2, 1 case). ILD incidence was 23.1% in the present study, which is similar to that reported previously in a subset of Japanese patients treated with T-DXd (22.2%) [10]. The median time-to-discontinuation of T-DXd treatment due to ILD was 5.3 months in the present study, which is consistent with the 5.4 months reported in a previous pooled analysis [11].

In previous studies, multivariate factors contributing to OS included a BM diagnosis within 6 months of the date of metastatic recurrence diagnosis, HER2+ status, HR+ status, age, ECOG PS, and asymptomatic BM [12, 13]. In this study, the presence of LMC, components of GPA scoring (age, number of BM, metastasis except brain), ECOG PS, and other background factors (HER2 immunohistochemistry, estrogen receptor status, steroid use at the time of T-DXd administration, surgery, and line number) were not found to be prognostic factors of OS.

This study has some limitations, including those inherent to the retrospective design. Reporting bias is possible as the presence/absence of BM was determined by the investigator. LMC was diagnosed on imaging by ICR, and we did not confirm the presence of tumor cells in spinal fluid. The PFS may have been overestimated as the frequency of imaging evaluation was not specified. The generalizability of the findings is limited to Japanese patients.

In conclusion, the updated results of this retrospective chart review show that T-DXd has promising effectiveness in heavily pre-treated HER2+ MBC patients with BM and LMC. As late-onset ILD can also occur, it is important to conduct long-term monitoring to ensure early detection of ILD.

## Supplementary Information

Below is the link to the electronic supplementary material.

Supplementary file1 (DOCX 31 KB)

## Data Availability

The datasets used in the current analysis are available from the corresponding author upon reasonable request.
