# Correction: Trastuzumab deruxtecan for the treatment of patients with HER2-positive breast cancer with brain and/or leptomeningeal metastases: an updated overall survival analysis using data from a multicenter retrospective study (ROSET-BM)

**DOI:** 10.1007/s12282-024-01632-z

**Published:** 2024-09-20

**Authors:** Takahiro Nakayama, Naoki Niikura, Takashi Yamanaka, Mitsugu Yamamoto, Kazuo Matsuura, Kenichi Inoue, Sachiko Takahara, Hironori Nomura, Shosuke Kita, Miki Yamaguchi, Tomoyuki Aruga, Nobuhiro Shibata, Akihiko Shimomura, Yuri Ozaki, Shuji Sakai, Daisuke Takiguchi, Takehiko Takata, Armin Bastanfard, Kazuhito Shiosakai, Junji Tsurutani

**Affiliations:** 1 Department of Breast and Endocrine Surgery, Osaka International Cancer Institute, Osaka, Japan; 2 Department of Breast Oncology, Tokai University School of Medicine, 143 Shimokasuya, Isehara, Kanagawa 259-1193 Japan; 3 Department of Breast Surgery and Oncology, Kanagawa Cancer Center, Kanagawa, Japan; 4 Department of Breast Oncology, Hokkaido Cancer Center, Hokkaido, Japan; 5 Department of Breast Oncology, Saitama Medical University International Medical Center, Saitama, Japan; 6 Division of Breast Oncology, Saitama Cancer Center, Saitama, Japan; 7 Department of Breast Surgery, Kitano Hospital, Osaka, Japan; 8 First Department of Surgery, University of the Ryukyus Hospital, Okinawa, Japan; 9 Department of Medical Oncology, National Cancer Center Hospital, Tokyo, Japan; 10 Department of Breast Surgery, JCHO Kurume General Hospital, Fukuoka, Japan; 11 Department of Breast Surgery, Tokyo Metropolitan Cancer and Infectious Diseases Center Komagome Hospital, Tokyo, Japan; 12 Cancer Treatment Center, Kansai Medical University Hospital, Osaka, Japan; 13 Department of Breast and Medical Oncology, National Center for Global Health and Medicine, Tokyo, Japan; 14 Department of Breast Oncology, Aichi Cancer Center, Aichi, Japan; 15 Department of Diagnostic Imaging and Nuclear Medicine, Tokyo Women’s Medical University, Tokyo, Japan; 16 Oncology Medical Science Department I, Daiichi Sankyo Co., Ltd., Tokyo, Japan; 17 Data Intelligence Department, Daiichi Sankyo Co., Ltd., Tokyo, Japan; 18 Advanced Cancer Translational Research Institute, Showa University, Tokyo, Japan

**Correction: Breast Cancer** 10.1007/s12282-024-01614-1

In Table 2 of this article, the number of cases, ‘N = 114’ in the column head “Updated results; …” should have read ‘N = 104’.

The original article has been corrected.

