# Supplementary material for: Trastuzumab deruxtecan for the treatment of patients with HER2-positive breast cancer with brain and/or leptomeningeal metastases: an updated overall survival analysis using data from a multicenter retrospective study (ROSET-BM)
Source: Breast Cancer. 2024 Aug 12;31(6):1167–75. doi: 10.1007/s12282-024-01614-1 (PMC11489233; doi:10.1007/s12282-024-01614-1)
Supplement: Supplementary file 1 — Supplementary file1 (DOCX 31 KB) [file 12282_2024_1614_MOESM1_ESM.docx]

**Supplementary Materials**

**Journal name: Breast Cancer**

**Trastuzumab deruxtecan for the treatment of patients with HER2-positive breast cancer with brain and/or leptomeningeal metastases: An updated overall survival analysis using data from a multicenter retrospective study (ROSET-BM)**

Takahiro Nakayama, Naoki Niikura, Takashi Yamanaka, Mitsugu Yamamoto, Kazuo Matsuura, Kenichi Inoue, Sachiko Takahara, Hironori Nomura, Shosuke Kita, Miki Yamaguchi, Tomoyuki Aruga, Nobuhiro Shibata, Akihiko Shimomura, Yuri Ozaki, Shuji Sakai, Daisuke Takiguchi, Takehiko Takata, Armin Bastanfard, Kazuhito Shiosakai, Junji Tsurutani

Corresponding author: Naoki Niikura, MD, PhD, Professor

Affiliation: Department of Breast Oncology, Tokai University School of Medicine, Kanagawa, Japan

E-mail: nniikura@tokai.ac.jp

**Supplementary Table 1** **Cox regression analysis of OS from first administration of T-DXd (total population)**

|  | **Univariate (*N* = 104)** | | | |
| --- | --- | --- | --- | --- |
|  | **Event / *N*** | **HR** | **95% CI** | ***P* value** |
| Age, years |  |  |  |  |
| ≥60 vs <60 | 14/42 vs 29/62 | 0.64 | 0.34–1.20 | 0.164 |
| HER2 IHC |  |  |  |  |
| 3+ vs 2+/ISH+ | 32/84 vs 10/18 | 0.58 | 0.29–1.19 | 0.136 |
| Estrogen receptor status |  |  |  |  |
| Negative vs positive | 21/44 vs 22/59 | 1.19 | 0.66–2.17 | 0.562 |
| Steroid use at the time of T-DXd administration |  |  |  |  |
| Absence vs presence | 36/89 vs 7/15 | 0.72 | 0.32–1.62 | 0.429 |
| Surgery for primary breast cancer |  |  |  |  |
| De novo vs recurrence | 11/33 vs 32/71 | 0.69 | 0.35–1.36 | 0.280 |
| ECOG PS |  |  |  |  |
| 0–1 vs ≥2 | 33/81 vs 6/16 | 0.88 | 0.37–2.11 | 0.776 |
| Number of BM |  |  |  |  |
| 1 vs ≥2 | 10/18 vs 27/72 | 1.45 | 0.70–3.00 | 0.318 |
| LMC |  |  |  |  |
| Without LMC vs LMC | 34/79 vs 6/19 | 1.48 | 0.62–3.52 | 0.379 |
| Line number |  |  |  |  |
| 0–2 vs ≥3 | 11/25 vs 32/79 | 1.01 | 0.51–2.01 | 0.976 |
| Metastasis except brain |  |  |  |  |
| Negative vs positive | 0/4 vs 4/100 | 0.00 | 0.00–NA^a^ | 0.990 |

Stepwise analysis.

^a^Factors with fewer than 10 patients in one group (metastasis except brain; negative vs positive) were not analyzed.

*BM* brain metastasis, *CI* confidence interval, *ECOG PS* Eastern Cooperative Oncology Group performance status, *HER2* human epidermal growth factor receptor 2, *HR* hazard ratio, *IHC* immunohistochemistry, *ISH* in situ hybridization, *LMC* leptomeningeal carcinomatosis, *NA* not analyzed, *OS* overall survival, *T-DXd* trastuzumab deruxtecan.
